# Sharp Injuries Among Medical Students

**DOI:** 10.5539/gjhs.v7n5p320

**Published:** 2015-03-30

**Authors:** Iman Ghasemzadeh, Mitra Kazerooni, Parivash Davoodian, Yaghoob Hamedi, Payam Sadeghi

**Affiliations:** 1 Infectious & Tropical Diseases Research Center, Hormozgan University of Medical Sciences, Bandar Abbas, Iran; 2 Student Research Committee, Hormozgan University of Medical Sciences, Bandar Abbas, Iran; 3 Department of Parasitology, Hormozgan University of Medical Sciences, Bandar Abbas, Iran

**Keywords:** needlestick, sharp injuries, epidemiology, students

## Abstract

**Introduction:**

Sharp injuries threaten the health of healthcare employees. They cause the transmission of many diseases such as hepatitis B and C, AIDS, etc., which can increase the associated costs associated with them. The aim of this study was to investigate the frequency of sharp injuries among the students of Hormozgan University of Medical Sciences.

**Method:**

This cross-sectional study was conducted during 2012-2013 in Hormozgan University of Medical Sciences, IR Iran. The target population consisted of the medical, nursing, midwifery, operating room technician, and medical laboratory students in the 2012-2013 academic year. Census sampling was conducted, and accordingly, 500 students participated in the study Data was collected using modified questionnaire of the University of San Diego’s injury report form. The collected data were entered into SPSS V.19 and analyzed using descriptive statistical tests.

**Findings:**

Finally 377 students (75.4%) returned the questionnaire. Among the studied students, 184 students (39.3%) had had sharp injuries. The frequency of damaging Vein puncture was the most common mechanism of injury

**Discussion and Conclusion:**

The prevalence of sharp injuries is high among students which can increase the risk of disease and its subsequent risks, and thus, increase the cost and stress among students. It seems that holding workshops and increasing students’ awareness and skills to face these risks can be effective in mitigating them.

## 1. Introduction

Needlestick and sharps injuries threaten all people employed in the healthcare system and has caused much attention in promoting job security of healthcare system personnel (Heidari & Shahbazi, 2010). Needlestick and sharps injuries (NSIs) are defined as “penetrating wound with an instrument that is potentially contaminated with body fluids of another person.” According to the United States National Institute for Occupational Safety and Health (NIOSH), needlesticks are injuries caused by needles such as hypodermic needles, blood collection needles, sterile intravenous needles, etc. (Galougahi, 2010).

Sharps injuries can expose healthcare workers to blood-borne infections such as HIV, hepatitis C, B, toxins and many other diseases (Bernard et al., 2013). In the United States most centers for diseases control and prevention report about 385,000 sharp injuries in healthcare centers in 2000 (Wilburn, 2004) Various studies suggest the transmission of at least 20 organisms in sharp injuries (Saleem et al., 2010). Economic costs imposed by the treatment and the consequent stress are the other complications of sharp injuries (Zanni & Wick, 2007). Sharp injuries prevalence is also significant in some countries, including US. Job stress during performing duty is reported to be a contributor factors in this regard (Makary et al., 2007).

Needlesticks and sharp injuries case reports are an important factor in the improvement of protection against this problem (Thomas & Murray, 2009). A number of diseases which transferring by needle and sharp is HBV, HCV, HIV et al. (Khurl-Bulos et al., 1997) Low rate of skin sharp injury reports is a great danger for healthcare employees and workers which can be due to the lack of awareness (Shokuhi et al., 2012). Therefore, determining the level of exposure to these risks and planning about them can mitigate their respective risk and injuries.

Given the importance of the issue and the lack of similar studies in the Hormozgan province and among its students, sharp injuries and some underlying and predisposing factors among the students of Hormozgan University of Medical Sciences were investigated. The results can used to appropriately plan to reduce these injuries and their causes.

## 2. Method

This cross-sectional study was conducted in 2012-2013 in the allied health, nursing, midwifery, and medicine departments of Hormozgan University of Medical Sciences. Hormozgan province is located in southern of Iran and north of Persian gulf and Oman Sea (Vatandoost et al., 2004). The target population consisted of the medical, nursing, midwifery, operating room technician, and medical laboratory students who were intern in the university hospitals of Bandar Abbas (Shahid Mohammadi Hospital, Children’s Hospital, and Doctor Shariati Hospital) in the 2012-2013 academic year. Census sampling was conducted, and at the end, 500 students participated in the study. Inclusion criteria included being enrolled in the 3^rd^ to 14^th^ semester, and the consent to participate in the study. The exclusion criteria included interns in medicine and other disciplines who were not practicing at the moment, and lack of cooperation and proper completion of the questionnaire. Data was collected using modified questionnaire of the University of San Diego’s injury report form to information network of exposure prevention and using other articles (Galougahi, 2010). The questionnaire included demographic characteristics (age, sex, discipline, semester, hospital, the workplace) as well as the characteristics related the study (cause of the incident, time of incident, injured member, cause of injury, and three questions related to engineering, if any), and section related to the explanation of how the incident had happened. A section was also provided for participants to submit their proposals. The confidentiality of information was explained to the students. They were assured that the information was going be used in a research work. The collected data were entered into SPSS V.19 and analyzed using descriptive statistical tests in our study p-value is 0.05.

## 3. Findings

A total of 500 questionnaires that were distributed among the students, and 377 students (75.4%) returned the questionnaire. Among them, 181 (48%) were men, and 196 (52%) were women. Their mean age was 23.63±1.72 with maximum of 20 years, and maximum of 29 years. The mean semester in which they were enrolled was 8.8±2.9. The frequency by different disciplines was as follows: 40 (10.6%) operating room technician; 44 (11.7%) medical laboratory; 50 (13.3%) nursery; 127 (33.7%) medicine. The place of internship at the time of data collection was as follows: 120 (31.8%) in the patients’ room (hospital), 43 (11.4%) in the delivery room, 32 (8.5%) in operating room, 7 (1.9%) in the intensive care unit (ICU/CCU), 44 (11.7%) in the laboratory, and 131 (34.7%) in the emergency department. Among the participants, 365 students (96.8%) had the history of receiving hepatitis vaccine, and 5 students (1.3%) had not received hepatitis vaccine, and 7 students (1.9%) had not received it completely.

Among the studied students, 184 students (39.3%) had had sharp injuries. The frequency of damaging instruments is shown in Table 1.

**Table 1:** The frequency of damaging instruments

Instrument	Frequency
Needle	67 (45.3%)
Suture needle	46 (31.1%)
Angiocath	29 (19.6%)
Scalpel	2 (1.4%)
Used ampule	2 (1.4%)
Shaving blade	1 (0.7%)
Test tube	1 (0.7%)
Total	184 (39.3%)

Vein puncture was the most common mechanism of injury (24.3%). The other causes were as follows: drawing arterial blood in 20.3%, injections in 7.4%, replacing the IV line in 7 students (4.7%), suturing in 36.5%, and other causes in 9 students (6.1%). The reasons for these incident were as follows: 82 (55.4%) due to distractions, 24 (16.2%) due to a busy shift, 21 (14.2%) due to forgetting, 10 (6.8%) due to the restlessness of the patient, and 10 (6.8%) due to excessive fatigue and lack of education. The timing of the accidents was as follows: in 118 (79.9%) cases, during work; in 11 (7.4%) cases, when disposing of the sharp instruments; in 4 (2.7%) cases, when putting the cap on the sharp instruments; in 10 (6.8%) cases, when disposing of the sharp instruments into the special bin; and in 5 (3.4%) cases, when separating parts of a sharp instrument. The frequency of the injured members was as follows: in 135 (91.2%) cases, fingers; in 11 (7.4%) cases, hand; in 1 (0.7%) case, head and face; and in 1 (0.7%) case, leg. The frequency of the place of the occurrence of the event was as follows: 63 (42.6%) cases in the emergency department; 53 (35.8%) cases in the patients’ rooms; 17 (11.5%) cases in the delivery room; 14 (9.5%) cases in the operating room; 1 case in the laboratory; and 7 (4.7%) cases in CCU/ICU. Among the students, 352 (93.3%) students said that no special equipment is available to prevent sharp injuries in the department. Forty-five (11.9%) students stated that protective equipment was used all the time, 109 (28.18%) students stated that protective equipment was used partially, and 423 (58.8%) students stated that protective equipment was not used at all. 371 (97.9%) students had access to the Safety Box. Eighty-five (57.4%) men and 63 (42.6%) women had had an incident. No significant relationship was observed between gender and the incidence of injury (p-value=0.004). No significant relationship was observed between discipline and the incidence of injury (p-value=0.089). A significant relationship was observed between the enrolled semester and the incidence of injury (p-value <0.001) so that the chance of injury was higher in senior students. A significant relationship was found between workplace and the incidence of injury. Sixty-three (42.6%) of the injured students were interns in the emergency department (P-value <0.001).

## 4. Discussion

Injuries caused by blood-contaminated needles can cause serious and fatal complications and are hazardous occupational injuries. They can cause transmission of dangerous diseases such as hepatitis B, C and HIV. More than 20 pathogens are transmitted through needles and sharp instruments (Baghcheghi et al., 2011). The purpose of this study was to investigate the frequency of sharp injuries during training in students of Hormozgan University of Medical Sciences.

In our study, 39.3% of participants had been injured by sharp instruments. In a study in 2003 in Missouri, USA, the frequency of needlestick (NS) incident was reported to be 30% (Patterson, Novak, Mackinnon, & Ellis, 2003). Studies in Lima and Peru reported that 46.7% of the students had been at least once in contact with blood or body fluids during the first nine months of 2012 (Díaz & Cadena, 2002).

Naing (Naing, Zulkifli, & Kamaruzzaman, 1995) estimated the prevalence of NS is about 38.3%. 30% of medical students in Washington (Patterson et al., 2003) and 61.9% of nursing students in Taiwan (Shiao et al., 2002) had at least an incident of NS, and most of the injuries had occurred in the patients’ room. In the present study, patients’ room (35.8%) was the second most common place of injury.

In a study conducted by Wicker (Wicker, Nürnberger et al., 2008), 58.8% of the students stated that they had at least one incident of NS during their studies. Among them, 37.2% reported that had been injured more than once. English (1992) studied the staff of a hospital in Washington in 1992 and reported that nurses (45.8%) had more incident of NS than any other hospital employees. A study in China (Smith et al., 2004) reported that 95% of the nurses in a hospital had had an incident of NS. A study in Iran (Askarian & Ghavanini, 2001) reported that 31.7% of the anesthetists had had an incident of NS. A study conducted on 688 medical, dentistry, nursing, midwifery students of Shiraz University of Medical Sciences (Askarian & Malekmakan, 2006) showed that 71.1% of the participant had had at least one incident of NS which was very high compared to other countries. In a study conducted by Norsayani (Norsayani & Noor Hassim, 2003), the prevalence of NS was reported to be 14.1% which was lower compared to other studies.

In the present study, the most common instruments that had caused injuries were needles, suture needles, and angiocath. In a study conducted by Shahbazi (Heidari & Shahbazi 2010), the most common instruments were suture needles and scalpel. Rakhshani (Rakhshani et al., 2009) showed the most common instruments were needles (55.4%). In a study in China (Wang et al. 2003) conducted by Wang (2003), conventional syringe needle were the most common instruments after intravenous needles which is consistent with the present study. In a study conducted at Shiraz University of Medical Sciences (Askarian & Ghavanini, 2001), as well as a study conducted by Rakhshani (Rakhshani et al., 2009), the most common injury situations were drawing blood and IV injection which had the frequencies of 28.6% and 56.4%, respectively.

Noohi (Noohi et al., 2010) reported that the most common cause of injury was syringe needle. It was also reported that removing the needle’s cap (23.8%), recapping needles (23.8%), and drawing medicine (23.8%) were the activities leading to this injury. The results of Norsayani (Norsayani & Noor Hassim, 2003) and Aaghdoust (Aghadoost et al., 2007) were consistent with these results. This can be important since 67.4% of students recap needles after using them.

Our results show that a large proportion of NS incidents (42.6%) occur in emergency department, while Askarian (Askarian & Ghavanini, 2001) reported that the most commons place of NS incidents is in patients’ room. Aghajanloo (Aghajanloo et al., 2007) reported the otolaryngology and surgery units are the most commons place of NS incidents, and Siddique et al. (Yang et al., 2004) suggested that emergency department is the most hazardous places regarding the NS incidents in students. Higher risks of injury in emergency department can be justified since this department us highly active, and various processes are carried out by students and trainees. Crowdedness, stress, time limitation, and the patients’ critical condition can be influential in this regard. A significant relationship was observed between gender and the possibility of injuries. Males were more prone to injuries. This result was consistent inconsistent with the results of Aghajanloo (Aghajanloo et al., 2007), Shahbazi (Heidari & Shahbazi, 2010) and NgYW (Ng & Hassim 2007). This result may be due to failure to comply with protective protocols in men. In our study, 365 (96.8%) students had received hepatitis vaccine. In Muhammadnejad’s (Mohammadnejad et al., 2009) and Mohammed’s (Mohammadi et al., 2011) studies, vaccination coverage rates were 82.4% and 96.3%, respectively. In Taiwan, 16% of the nursing students had not received hepatitis vaccine (Shiao et al., 2002). This rate was higher among Turkish students where 32.4% were not vaccinated (Ayranci & Kosgeroglu, 2004). This finding shows an appropriate conditions regarding immunity against hepatitis among students in our study. A significant relationship between the prevalence of NS and the enrolled semester so that injuries were more frequent in more senior students, which is consistent with the results of Aghajanloo (Aghajanloo et al., 2007). Norsayani (Norsayani & Noor Hassim, 2003) (20.9%), and Smith and Leggat (2005) suggested that students in the final year of their studies are more prone to injury because they have more clinical activities which increases the possibility of injury

## 5. Conclusions

The incidence of needlestick injuries caused by needles is high among students. On the other hand, some of the students exposed to these injuries do not act appropriately. Many of these injuries can be prevented by proper education regarding protective measures during administration of medications and intravenous fluids and the use of safe instruments rather than conventional instruments. Further trainings on the prevention of these injuries and the consequent measures should be provided for the students. Rate of occupational exposure in different groups is undesirable compared to similar studies. Therefore, recording the cases of occupational exposures and their causes and attending to the personnel prone to exposure can help prevention of exposure to blood and communicable diseases. In general, and given the results, the following is recommend: Educational workshops on blood-borne diseases and sharp injuries, relevant training (if possible), and the use of modern and available instruments to mitigate injuries. It is also recommended that further studies be conducted on the awareness of students about infectious diseases to be a basis for planning measures regarding this issue. Such proposal has already been presented at our university, however, they are limited.

**Limitations**

The limitations of this study include the absence of some students in particular days, failure of some students to complete the questionnaire, and the exclusion of dentistry students from the study.
